# How the cost of veterinary care impacts the wellbeing, learning and practice of early career vets

**DOI:** 10.1002/vetr.4597

**Published:** 2024-09-11

**Authors:** Rachel Williams

**Affiliations:** ^1^ Cardiff Business School, Cardiff University Cardiff UK

**Keywords:** cost of care, pragmatic care, spectrum of care, stress, veterinarian, veterinary fees

## Abstract

**Background:**

Investigations by the Competition and Markets Authority into the veterinary sector have highlighted the cost of veterinary care. This paper examines the changing attitudes of early career vets towards these costs.

**Methods:**

Ninety‐seven semi‐structured interviews were held with 25 vets as part of a 2.5‐year longitudinal study. An inductive approach to analysis was adopted with flexible thematic analysis being undertaken using NVivo 12.

**Results:**

High veterinary care costs create a financial burden for clients and an emotional burden for vets. Vets felt unprepared to deal with restricted client budgets and were unsupported by their practices in relation to charging. When owners could not afford treatment, vets had fewer opportunities to perform procedures and practise their skills. Eventually, vets distanced themselves from their clients’ circumstances to prioritise their own wellbeing and began to value their expertise and charge appropriately.

**Limitations:**

The small sample size and emphasis on graduates of UK universities limit the generalisability of the findings.

**Conclusion:**

This paper highlights the impact of veterinary care costs on early career vets’ wellbeing, practice and learning opportunities and advocates a ‘spectrum of care’ approach to veterinary education. It also encourages practices to engage with vets regarding charging behaviours.

## INTRODUCTION

The decision by the UK Competition and Markets Authority (CMA) to instigate a market investigation into the veterinary sector has highlighted the cost of veterinary care.^1^ This has intensified the media narrative that some vets prioritise profit over animal welfare^2^ and led to concerns by the BVA and RCVS regarding increases in abusive behaviour towards vets.^3^, ^4^

Cost‐driven decision making creates significant challenges when vets are new to the demands of clinical practice. Recent news articles concerning the CMA investigation have provided useful context for the topic.^5^ Most notably, an article published in the Guardian^2^ highlighted the impact of costs on client decision making and outcomes for pets and purported that vets recommend unnecessary diagnostic tests. Meanwhile, studies examining stress within the vet profession have included the cost of care as a contributory factor.^6^, ^7^ However, little has been written about early career vets' perceptions of the cost of care, although this may have consequences for standards of care, practice management and vet retention.

The cost of care influences an owner's choice of treatment. Indeed, in the 2019 RCVS survey,^8^ vets expressed concern that high care costs were leading to owners, even those with insurance, being priced out of treatment. This finding supports a study by Rah and Choi,^9^ which suggested that high veterinary costs contribute to the abandonment of companion animals in lower socioeconomic status communities.

Another consequence of high fees is an increase in the level of stress experienced by vets,^6^, ^7^ particularly when required to perform euthanasia due to financial reasons.^10^ Kondrup et al.^11^ highlighted the ethical dilemma faced by vets when presented with financially constrained treatments and suggested that individual differences may influence how intensely stress is experienced. Other studies suggest that female vets experience greater stress than male vets when clients cannot afford treatment. Female vets may believe more strongly in the human–animal bond,^12^ be more inclined to subsidise comprehensive treatment^11^ and be more emotionally affected by a decision to carry out euthanasia.^13^ It is therefore possible that they may be disproportionately impacted by increases in fees, although individual differences are likely.

Kipperman et al.^14^ highlighted that new vets, in particular, may find performing euthanasia stressful, particularly when this is due to convenience or inability to pay; however, their study does not define ‘convenience’. They identified an emphasis on animal advocacy among veterinary students, whereas experienced practitioners focused more on client needs. The authors suggested that, as vets gain experience, they become more client focused and find that the process of euthanasia for financial or convenience reasons becomes less stressful. However, other authors^11^, ^13^ suggested that the degree of stress caused by these cases is not related to the vet's level of experience, although a higher frequency of encounters may increase dismissiveness towards the owner's financial difficulties.

Stress may lead to employee turnover, with a survey by Hagen et al.^15^ finding that work–life balance and poor management were common reasons for vets to leave their jobs. The BVA Good Workplaces initiative and Great Workplace accreditation schemes^16^ map out the factors that help employees thrive in veterinary practices. These factors include supportive cultures and compassionate leadership; both valuable resources when vets are faced with economic challenges.

How well vets cope with owners with restricted budgets may be influenced by their professional identity. Armitage‐Chan and May^17^ found that new vets who developed a ‘diagnostic focused’ identity drew satisfaction from demonstrating technical competence in their work but were frustrated when faced with barriers such as their client's inability to pay. Vets who displayed a ‘challenge focused’ identity were more likely to see financial barriers as interesting challenges and gain satisfaction from treating animals regardless of the context. This therefore suggests that, alongside the need for veterinary students to be trained in business skills and client communication,^11^, ^18^, ^19^ veterinary education should focus beyond providing a gold standard of treatment. Warman et al.^20^ support a spectrum of care (SoC) approach, proposing that a variety of treatment pathways may offer equally acceptable outcomes, a view supported by the BVA.^21^ Teaching that supports this approach should equip students to treat patients effectively, taking into account their own abilities, the facilities available and the client's budget. Similarly, Manktelow^22^ highlighted the benefits of adopting a pragmatic case management model where the owner's resources are discussed openly and a flexible approach to treatment is adopted. This model may have benefits for the wellbeing of both practice teams and patients.

This study is part of a wider project examining the experiences of early career vets. It builds on existing literature to address the research question, ‘How does the cost of veterinary care impact the perceptions and practice of early career vets?’

## METHODS

A qualitative, longitudinal approach was used to examine the experiences of 25 veterinary graduates and identify changes over time. Ninety‐seven semi‐structured interviews were conducted in four phases: vets were interviewed between June and September 2021 (before starting work as a qualified vet), after 6–8 months in practice, after 12–18 months and, most recently, between December 2023 and April 2024. Between 23 and25 vets participated in each phase. Twenty‐two of the vets were female and three were male. Initially, 13 of the vets worked in small animal practices, with the remaining working in farm and mixed practices. Eighteen worked for practices owned by corporate organisations and seven worked in independent practices.

Participants were recruited via the closed Facebook group Vet Voices UK. Purposive sampling was used, with vets due to graduate from a UK vet school that year (2021) being invited to participate. At least one participant was recruited from each UK vet school. All participants received information about the study before signing a consent form.

The author, a Reader in Human Resource Management, conducted all interviews via Zoom, with only the participant present. The interviews lasted between 40 and 70 minutes. Semi‐structured interviews provided scope for participants to identify their own important issues. The impact of fee increases was not a planned question; however, several participants mentioned fees when discussing the challenges they faced. Therefore, in the fourth interview, the participants were asked ‘Do you believe that the fees your practice charge are fair? The participants received questions in advance for the third and fourth interviews to allow time for reflection. The interviews were audio recorded and transcribed.

An inductive approach to analysis was adopted, with flexible thematic analysis being undertaken by the author as described by Braun and Clarke.^23^, ^24^ Transcripts were read and reread, codes such as ‘preparedness for practice’, ‘worst experience’ and ‘level of support’ were identified and themes such as ‘cost of care’ were generated from these codes. NVivo 12 was used to record the process. The author works outside the veterinary profession; therefore, the data were interpreted through the lens of an outsider. Braun and Clarke^23^ suggest that meaning is situated in the researcher's experience and their subjectivity ‘sculpts the knowledge produced’.

## RESULTS

Where quotes have been presented, pseudonyms have been used, and the interview phase (first to fourth) is indicated.

### Attitudes towards fees

A significant finding was that participants’ attitudes towards charging clients became more nuanced as they gained experience. Initially, few vets considered the cost of care, possibly due to limited client interactions at university, exacerbated by the COVID‐19 pandemic and the consequent restrictions on students gaining real‐world practical experience. Consequently, when they started practising, some were shocked by the level of charges and how a client's inability to pay impacted treatment.

‘I didn't expect … the constant price increases … because it's then managing the clients when they've collected a prescription one month and they're back this month to collect it and it's £20 more … it's more like, just business‐y and like, I'm not interested in business, so that has been kind of harder or more challenging than expected’. (Anna, third interview)

Several small animal vets described subversive behaviour, such as ‘forgetting’ to charge for items or charging a lower fee. Often, this was when a treatment had been quick, and they believed the fees were not justified.

‘I have been known to occasionally, you know, not charge for something here or maybe charge a reduced fee for here, but technically I shouldn't be doing that’. (Sally, third interview)

After 2 years, vets’ views on charges started to vary. Some vets worried that price increases were impacting patient welfare, driving clients to cheaper practices and limiting opportunities to practise procedures and learn skills. Vets voiced these concerns but did not feel heard.

‘The [x‐ray] price has practically doubled overnight and, since then, we've got none … Like, you give them the quote and you can see the shock in their face, and they walk away, never to return, and you're kind of like, yeah, I knew that would happen’. (Alex, fourth interview)

‘[We've been told] if we don't make more money, people are going to lose their jobs … we've got too many vets … the reason we're not doing as much work is because people can't afford it … and so one of the ways as to how to resolve that was by putting prices up, and it's just completely counterproductive, … I put a lot of animals to sleep, purely because of costs’. (Bethan, fourth interview)

Many vets were initially concerned about vet fees, but after 2 years, some accepted the charges and began to value their expertise. Some gave credit to their mentors for this change, whereas others reached this view independently. They did not agree with all charges but were beginning to feel that some were justified.

‘Sometimes when I collect like a cytology sample, I was thinking … “I won't charge them” but now, I'm just like, “Yes, I'm going to charge you because that's 15 minutes of my time trying to look through the slides and then call you back”, so, yeah, I would say that I'm a lot better in charging appropriately’. (Terri, third interview)

Some vets also became less emotionally involved with clients. They were frustrated with those who had acquired pets without understanding the ongoing costs and demonstrated less sympathy for some owners.

‘I think, well, you chose to have a pet. Like, you should have known that you should put money away for these reasons, like having a car’. (Lilly, third interview)

Finally, a few vets explained that they had started to charge clients correctly because they realised that undercharging clients caused problems for colleagues and increased client complaints.

‘People are going to complain about money, whether you charge them correctly or not … it makes it really rubbish for other people if, like for the first consult, someone didn't charge it properly, and then you go ahead and charge it’. (Anna, fourth interview)

In contrast, very few farm vets believed that their practices charged excessive fees. They understood that farmers felt under pressure to save money but considered fees to be justifiable business expenses.

‘I think that's just the frustration of the low margins in the farming side … I think generally, they recognise that we provide a good enough service for the money we charge. We definitely don't get the stick like small animal vets do’. (Katie, fourth interview)

It was also notable that farm practice fees were often lower than those charged by small animal practices. One participant described being rung by a farmer with a request to perform a caesarean on his dog because he had been quoted £3000, whereas their practice charged £500 for a caesarean on a cow.

‘500 quid is a lot of money, don't get me wrong, but I think it's justifiable’. (Grace, fourth interview)

### Stress

Another area of interest has been the participants’ concern at the cumulative impact of the increasing costs of veterinary care, pet insurance and the cost of living on their treatment plans and the outcomes for patients. Vets reported the emotional impact of being unable to help animals solely due to cost.

‘People have cancelled their pet insurance, people don't have spare disposable income to spend … I just find it very difficult telling them how much it costs when I think it's massively overpriced … and it all drives the really upsetting days where you're literally putting healthy animals to sleep’. (Bethan, fourth interview)

One participant described an occasion when owners were unable to afford to treat their puppy. As a 1‐year qualified vet, they struggled with the responsibility of supporting the owner and their colleagues and managing their own distress.

‘I had a 4‐month‐old puppy came in with a fracture, and the client was standing there with his pregnant wife, saying “Well I can't afford even the x‐rays” … unfortunately we have to explain … “Well look, I'm sorry … the only thing we can do is let her go”, and I think that's a really difficult situation to be in … it is emotional for everyone, receptionists were getting upset, the nurses were getting upset, obviously we didn't want to be in that situation either’. (Hannah, third interview)

This highlights how the financial burden for clients impacts the prognosis for the pet, creating an emotional burden for vets. However, vets can develop resilience, with some becoming removed from the emotion.

‘It almost feels like I've got into a mode where … there's like a little emotional switch that sort of turns off when it's going into the consult room’. (Sam, fourth interview)

‘And then afterwards, I literally stood in prep over the cat with a couple of my colleagues and I was like, “Does it ever scare you how little emotion you feel about some of these cases?” … I think it's normal within the profession. It's not normal within humans. But within the job that we do, you just get very used to stuff like that’. (Anna, fourth interview)

### Practice ownership

There were fewer differences between the experiences of vets employed by corporate and independent practices than anticipated. Most of the small animal vets, regardless of practice ownership, had observed price increases, but occasionally fees in independent practices were lower.

‘I'd say it's probably a quarter of the price to do a dental, and it means that we do so many more, and so many more animals have healthy mouths … which makes me happier’. (Emma, fourth interview)

A similar situation existed in relation to fee remission. Most fees were non‐negotiable, although a few, mainly independent, practices did allow their vets discretion to discount fees.

‘Animal welfare comes first … we're able to, you know, quote lower prices for people who need it or who have struggled to find care elsewhere, which is really nice’. (Billie, second interview)

There was little evidence to support media reports that vets promote unnecessary services. However, one participant reported experiencing pressure to maximise income, having moved from one independent practice to another. They were surprised at the focus on targets and sales in their new practice.

‘What I've realised now is that they chart your follow‐ups … you are encouraged to push to get people back into the clinic. … If you get them back in the door, you can talk about teeth, you can talk about supplements … but it's not in my nature to do that’. (Billie, fourth interview)

The media reports are therefore worrying because, as the BVA suggests, they may incite further aggression towards vets. One vet working in a referral practice highlighted how surprised they were at the negative attitudes they experienced.

‘I didn't realise that the public would kind of vilify vets as much as they do … I thought vets were quite respected … But it's as if people just think we're kind of money‐grabbers … and it's definitely not why anyone that I know is in the profession’. (Charlie, third interview)

### Spectrum of care

Throughout the study, vets have discussed the tensions they experience between the gold standard of care taught at university and the pragmatic approach often required in daily practice. They have learned to provide treatment within their client's budget, diagnosing conditions with limited diagnostic tools and also honing communication skills. The vets talked about learning to ‘read owners’ and do their best with the limited budget available.

‘You do learn in vet school like, gold standard … and then in the first few years, it was learning how to take that and do what you can with a budget … and now it's just … water it down even more’. (Ffion, third interview)

Although they did not use the term SoC, several vets indicated that they would have valued this teaching during their degree, particularly to help them accept that they cannot resolve all problems with their available resources. For example, one participant described their distress at not saving a dog,

‘I just went home and was just, like, crying, because I just felt really bad. And I just felt guilty, because I thought, if that's my job that's not good enough. But equally, I don't think I could have done any more’. (Catrin, second interview)

## DISCUSSION

Overall, the findings highlight how care costs impact a vet's ability to treat patients to the preferred standard and illustrate the distress that this can cause. However, they also shed light on the resilience and adaptability of new vets; although frustrated by charges, they adapt their practice to accommodate each client's budget. They have also developed an understanding of the tensions around billing. Although it is distressing when owners cannot afford treatment, the vets have learned to value their expertise and appreciate the benefit of charging appropriately.

### Change in attitude towards fees

One of the most startling findings was how ill prepared the vets were for charging clients. Many small animal vets were initially shocked by the level of fees and fee increases. Consistent with a study by Bachynsky et al.,^18^ the newly qualified vets described ‘forgetting’ to charge for items and being uncomfortable charging for short repeat consults. However, after 2 years many vets stopped discounting fees and believed that many charges were justifiable. This change was not observed in all participants, but many were adopting a more commercial approach. Although none were involved in management roles, the ethos of their practices was influencing their attitudes, and they appreciated how charging incorrectly increased client complaints.

There was also evidence, consistent with the findings of Kondrup et al.,^11^ that vets were becoming less sympathetic towards clients who could not afford treatment. Some were frustrated that animals suffered because owners did not investigate the cost of ownership before purchase, whereas others began to accept that many care costs were justified. In other cases, vets did not have the time or capacity to become emotionally involved. This raises the question of whether it is desirable for vets to become more distant in order to protect their wellbeing or if it is important for vets to feel empathy for both clients and patients.

As they gain experience, many vets accept the rationale for care costs but still have concerns about some charges. They express frustration that when they raise concerns, managers explain how to improve selling skills rather than acknowledging the loss of business due to clients’ inability to pay. This emphasises the need for managers to engage in discussions about the cost of care, both to educate early career vets and to understand their concerns. Merely enforcing the fee structure and sanctioning vets who charge incorrectly could lead to disengagement and potential employee turnover.

It was interesting that farm vets were more likely to feel that the fees charged were reasonable and to describe clients accepting these charges. This may be because the fees charged were lower than the equivalent fees charged by small animal practices or because they were accepted as inevitable business expenses. Farm vets believed they earned lower salaries than small animal vets, which could contribute towards the lower overheads.

### Links between fee level and treatment options

The 2019 RCVS survey indicated that costs were deterring owners from accessing veterinary care and preventing vets from providing their preferred treatments. Kipperman et al.^14^ highlighted how these consequences may lead to disillusionment and burnout. This is supported by the findings of this study, which suggest that performing euthanasia when clients cannot afford treatment is distressing due to conflict between vets’ personal values and their clients' wishes. Armitage‐Chan and May^17^ suggested that vets with a ‘diagnosis‐focused’ identity are more likely to feel distress when they cannot provide adequate treatment. Although the vets in this study did describe frustration, there appeared to be a continuum between the diagnostic‐focused and challenge‐focused identities. In most cases, the frustration at not being able to treat a condition was accompanied by a pragmatic outlook, and over time the vets learned to adapt their practice to accommodate each client's financial constraints. However, some vets still felt distress at not providing optimal care within the client's budget. This highlights a need for practices to provide a supportive culture where vets do not feel responsible when owners cannot afford gold standard care.

### Practice ownership

The study identified fewer differences between types of practice ownership than anticipated, although high fees were mentioned more often by vets in corporate practices. It was notable that some vets appeared to distance their own practice managers from financial decisions and emphasised how fees were set centrally and inflicted onto seemingly powerless individual practices. Sometimes, the managers appeared to adhere to corporate rules but undermined these by ignoring undercharging by vets. In other practices, vets were critical of managers for not opposing the increases.

Very few vets described pressure to sell additional products or treatments, and those that did expressed concern at the practice. This contradicts some press reports. Other vets stated that they would not work in practices where they needed to meet targets or up‐sell services, which suggests that in an employment market where experienced vets are scarce, the practice's priorities in relation to profit and fees could impact employee retention.

### Vet education

Finally, this study supports the shift in vet education towards an SoC approach. Several vets reported dissonance during early practice when they tried to apply the comprehensive diagnostic approach they were taught at university to their low socioeconomic status client base. The participants identified a need for training to help them adapt their practice to restricted client budgets. Several vets explained that they currently taught veterinary students as part of practice‐based curriculum initiatives and considered this type of teaching to be beneficial in preparing students for the realities of practice.

## LIMITATIONS

The main limitation of the study is sample size; therefore, the views expressed by the participants may not be representative of those held more widely in the profession. However, although the study is based on only 25 vets, many have changed practice during the study, and therefore, the 97 interviews relate to over 40 practices.

In addition, the target population for the study was graduates of UK veterinary schools, but many vets practising in the UK studied elsewhere. Indeed 36% of RCVS members have qualifications obtained outside of the UK.. Therefore, the study's findings may not be translatable to all vets practising in the UK.

## CONCLUSION

This study demonstrates how inflationary pressure on the cost of veterinary care leads to cost‐driven decision making by both vets and clients, highlighting the impact on the wellbeing of vets and the welfare of the animals they treat. Teaching an SoC approach as part of the veterinary curiculum may support newly qualified vets with the challenges posed by inflationary pressures and charging accurately.

## AUTHOR CONTRIBUTIONS

Rachel Williams was the sole author and conceived the project idea and design, carried out all interviews and data analysis, and drafted the paper.

## CONFLICT OF INTEREST STATEMENT

The author declares no conflicts of interest.

## ETHICS STATEMENT

The study was approved by the Cardiff University Research Ethics Committee.

## Data Availability

Due to the on‐going nature of this study, the data that support the findings are not currently shared in order to preserve the anonymity and confidentiality of the participants. It is the author's intention to make anonymised data available on request to researchers with legitimate motives at the end of the study.
